# A Systematic Review and Meta-Analysis of the Association Between Depot Medroxyprogesterone Acetate and Cerebral Meningioma

**DOI:** 10.3390/cancers18081252

**Published:** 2026-04-15

**Authors:** Lindy M. Reynolds, Rebecca C. Arend, Russell L. Griffin

**Affiliations:** 1 Department of Epidemiology, School of Public Health, University of Alabama at Birmingham, Birmingham, AL 35233, USA; lmreynolds@uabmc.edu; 2 Department of Obstetrics and Gynecology, Heersink School of Medicine, University of Alabama at Birmingham, Birmingham, AL 35233, USA; rarend@uabmc.edu

**Keywords:** medroxyprogesterone acetate, meningioma, contraceptive, progestin

## Abstract

**Background/Objectives:** Depot medroxyprogesterone acetate (dMPA) is a synthetic progestin commonly used for contraception. Recent studies have reported an increased association between dMPA exposure and diagnosis of cerebral meningioma. The current systematic review aims to provide a review of literature on the topic of dMPA and cerebral meningioma as well as conduct a meta-analysis by the duration of dMPA use. **Methods:** The current study presented a systematic review and meta-analysis of observational studies of dMPA and cerebral meningioma derived from PubMed, Web of Science, and Embase database searches for relevant studies published through February 2026. Odds ratios (ORs) and associated 95% confidence intervals were reported to determine the pooled effect of dMPA on cerebral meningioma diagnosis. Quality of evidence was assessed through the GRADE methodology. **Results:** Nine case-control studies and one cohort study were selected for review and analysis. The overall pooled OR was 2.78 (95% CI 2.20–3.52). This association was strongest for prolonged (i.e., ≥two-years) dMPA exposure (OR 3.49, 95% CI 2.35–5.18). GRADE analysis suggested a moderate quality of evidence. **Conclusions:** The results of this meta-analysis indicate that dMPA exposure is associated with an over two-fold increased odds of cerebral meningioma. This effect is consistent across studies and is stronger for prolonged dMPA exposure relative to short-term exposure, suggesting a dose–response effect. Clinicians should consider discussing with patients the cerebral meningioma risks associated with dMPA use when considering long-term birth control options.

## Simple Summary

Depot medroxyprogesterone acetate is an injectable, synthetic sex hormone often used for birth control. Recent research has reported that use of depot medroxyprogesterone acetate is associated with higher risk of cerebral meningioma, a type of (often benign) tumor found in the membranes surrounding the brain, particularly for longer duration use. The current study provides a review of the literature published to-date on the association between depot medroxyprogesterone acetate and cerebral meningioma as well as a meta-analysis to provide a single, pooled association for any exposure, short-term duration exposure, and prolonged exposure. The meta-analysis results suggest a higher risk of cerebral meningioma associated with depot medroxyprogesterone acetate use, particularly an over three-fold higher risk for use of two years or more.

## 1. Introduction

Medroxyprogesterone acetate (MPA) is a synthetic form of progesterone called a progestin that is used for a variety of gynecological and obstetric conditions (e.g., amenorrhea, endometrial hyperplasia, endometriosis) and is, for the current study, most notably used as a contraceptive [1]. It acts by binding to progesterone receptors in the body and preventing the release of gonadotropin-releasing hormone (GnRH). This decrease in the levels of GnRH then inhibits follicular maturation and increases the thickness of the cervical mucus, the effects of which prevent ovulation and decrease sperm mobility through the cervix. There are two forms of MPA, oral and injectable. The former requires consistent daily intake while the latter is an intramuscular injection that forms a depot within the muscle that provides a slow release of MPA lasting up to three months; as a result, the injectable form of MPA is known as depot medroxyprogesterone acetate (dMPA).

It is believed that progesterone receptors play an integral role in the formation of intracranial meningiomas, which are slow-growing, mostly benign tumors of the protective layer of the brain called the meninges [2,3,4,5]. Intracranial meningiomas are reportedly three times more prevalent among females than males [6,7] and have been observed to increase in size during periods of pregnancy when levels of progesterone are higher [8], particularly in healthy pregnancies [9]. Further, examination of meningiomas occurring during pregnancy revealed that all tumors expressed progesterone receptors while a lower expression frequency was observed among meningiomas that developed among women who were not pregnant [5]. A study examining progesterone receptor affinity among progestins observed that MPA has the fourth highest affinity behind progestins levonorgestrel, desogestrel, and nomegestrolacetate [10].

In 2024, a French study reported an over five-fold increased odds of cerebral meningioma among users of dMPA, an association that was driven by those who used dMPA for at least two years [11]. Since that time, a number of studies have been published that have examined the association between dMPA and cerebral meningioma among a variety of study populations; however, the reported associations range from weak to strong strengths of association due to differences in exposure duration, type of comparison group (e.g., active comparator, non-contraceptive user), study design, and prevalence of exposure. Recently, two review studies including the association between dMPA and cerebral meningioma have been published. The first of these studies provides a review with no meta-analysis, instead focusing on the implications of the South African population [12]. The second study [13] examined the association between cerebral meningioma and progestogens. While it did provide results of a meta-analysis, reporting a pooled odds ratio of 2.68 for dMPA, the study included only six dMPA-related studies, examined only any exposure to dMPA despite prior research finding that the association is particularly strong for prolonged dMPA exposure, and, in the pooled analysis, combined associations that included non-dMPA exposed individuals and non-dMPA users. Thus, the objective of this current systematic review and meta-analysis is to provide a description of the studies, assess the study quality, and address the limitations of the prior review studies by providing a pooled analysis of the association between dMPA and cerebral meningioma by duration of exposure (i.e., any, short-term, and prolonged) and type of comparator (i.e., non-exposed to dMPA, non-active, and active).

## 2. Materials and Methods

The current study was conducted following the Preferred Reporting Items for Systematic reviews and Meta-Analyses (PRISMA) guidelines (Supplementary Table S1); the review protocol was not prepared and not registered. A search was performed in PubMed, Embase, and Web of Science for articles published through February 2026. For each search, terms included medroxyprogesterone acetate, depot medroxyprogesterone acetate, progestin, and meningioma. Results were limited to scientific journal articles, review articles, and letters written in English. Further inclusion criteria included the study being an observational study design (i.e., case-control or cohort; disproportionality analyses using adverse event reports were excluded); the study including human subjects of female sex only; and a study that assessed the association between dMPA and the incidence of new cerebral meningioma through estimation of relative measures of association including odds ratios (ORs), risk ratios (RRs), or incidence rate ratios (IRRs) in addition to the associated 95% confidence intervals (CIs).

Once the final list of eligible articles was created, the titles and abstracts were reviewed by two of the authors (LR and RG) to determine which articles were to be included in the analytical sample for the current study. Reviewers were blinded to the journal, authors, and institution. There was a total of 45 disagreements between the reviewers; the reviewers discussed each of the studies, and a final decision was made. The full text of studies that were selected from initial review was then reviewed to determine final selection. For each selected study, using manual review, data was extracted on the study title, authors, year of publication, journal, study type (e.g., case-control, cohort), sample sizes, age range of participants, definition of dMPA exposure (i.e., source of exposure data, duration of exposure), confounders assessed, type of comparator group used, and relative measures of association. Type of comparator was defined as any non-dMPA exposed subject (which could include those who were taking a contraceptive other than dMPA), active comparator (i.e., a subject taking another contraceptive such as levonorgestrel or norethindrone), or non-active comparator (i.e., a subject taking no contraceptive). Exposure duration was categorized as any exposure regardless of duration, short-term duration (fewer than two years), or prolonged duration (two or more years). Of note, data from Griffin (2024) [14] were reanalyzed to make the exposure duration definition similar to studies that were subsequently published.

Quality of the final selected studies was determined through use of the JBI critical appraisal checklist for case-control studies by two authors (LR and RG) [15]. The checklist assesses the quality of studies across ten questions assessing the reliability of exposure and outcome measurement, matching methodology (for matched case-control and cohort studies), and analytical methodology. For each question, an answer of Yes, No, Uncertain, or Not Applicable may be chosen. The Grading of Recommendations Assessment, Development, and Evaluation (GRADE) methodology was used to determine the quality of evidence for the association between dMPA and cerebral meningioma [16,17].

Potential publication bias was assessed through visual inspection of contoured funnel plots of the point estimates and inverse of the standard error (i.e., precision) of the natural log of the odds ratio. As a study could be included in multiple pooled analyses (e.g., twice if the study provided short-term and prolonged exposure associations), the funnel plot was conducted among any exposure, short-term exposure, and prolonged exposure associations. An Egger’s regression test was used for formal testing of symmetry, with *p*-values < 0.05 suggesting asymmetry and potential publication bias.

Pooled analyses were conducted by each combination of type of comparator and duration of dMPA exposure. ORs were used as the measure of association for pooled associations across studies using both fixed and random effects analyses. If an OR was not reported for a given combination of exposure duration and comparator type, the study was excluded from that portion of the pooled analysis. Heterogeneity across studies was assessed utilizing an I^2^ and Q statistic. If heterogeneity was significant (*p* < 0.05) for a given duration category analysis, a random effects analysis utilizing the DerSimonian and Laird method [18] was used for pooled analysis; otherwise, a fixed effects pooled analysis utilizing the inverse variance method was used. In a post hoc sensitivity analysis, due to observed high heterogeneity, a leave-one-out analysis was performed using the weakest association to determine the effects of a study on the duration-specific effect and then separately for each combination of exposure type and comparator type.

Within each duration category, pooled ORs were computed for each sub-group of comparator type and for the overall duration. A forest plot was used to present results of the pooled analyses. An absolute measure of effect for dMPA exposure was calculated using the non-malignant cerebral meningioma incidence rate reported among females aged 35–54 by [19] and the overall pooled OR calculated from the meta-analysis. In a planned sensitivity analysis, pooled associations for each duration category were conducted using (regardless of comparator) the weakest association and, separately, the strongest association from each study. Microsoft Excel (Version 2508 Build 16.0.19127.20570) was used for the literature review and determination of included studies, and SAS v9.4 was used for all meta-analyses.

## 3. Results

### 3.1. Literature Search

**Figure 1:**
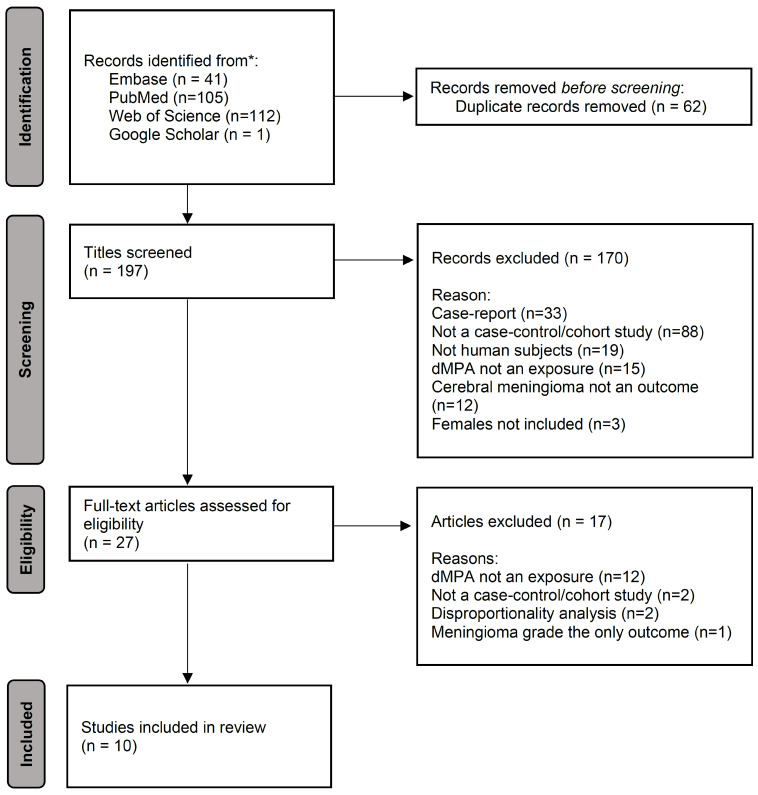
Flowchart of study screening, inclusion, and exclusion. * Includes a manuscript that was not indexed in the three selected services but was previously cited in a manuscript by the authors.

The literature search returned a total of 41 articles from Embase, 105 articles from PubMed, and 112 articles from Web of Science (Figure 1). In addition, one article [20] not indexed in the above services found, from a Google Scholar search, that a prior article by two of the current authors [21] was included. After removing 62 duplicates, 197 unique articles were selected for review for final consideration. Initial review involving titles of articles resulted in the exclusion of 170 studies, the majority of which (*n* = 88, 51.8%) were excluded due to not being a case-control or cohort study or case report (*n* = 33, 19.4%); the remaining studies were excluded due to not being a study involving human subjects (*n* = 19, 11.2%), not including MPA as an exposure (*n* = 15, 8.8%), not including cerebral meningioma as an outcome (*n* = 12, 7.1%), and not including females (*n* = 3, 1.8%). After review of the full text of the remaining 27, a total of 17 were excluded due to not including dMPA exposure (*n* = 12, 85.7%), not being a case-control or cohort study (*n* = 2, 14.3%), utilizing a disproportionality analysis design (*n* = 2, 14.3%), or examining only the association with meningioma grade (*n* = 1, 7.1%).

### 3.2. Study Characteristics

Of the ten selected studies [11,14,20,21,22,23,24,25,26,27]—which included nine case-control studies and one cohort study—a multitude (*n* = 8) were published between 2024 and 2026, though the first study to report an increased association with injection hormone exposure (particularly for prolonged use) was published in 2006 [22] (Table 1). A total of 139,672 cerebral meningioma cases were included across the studies; the number of participants exposed to dMPA ranged from seven to 88,667. Eight of the ten [11,14,20,21,23,25,26,27] identified dMPA exposure through medical record review or through pharmacy claims data; one study defined dMPA exposure from use of HCPCS and ICD-10 CM codes [24]; and one study defined dMPA exposure through the participant’s self-reported use [22]. All but one study defined cerebral meningioma diagnosis through use of ICD-9 or ICD-10 CM codes, specifically 192.1, 225.2, C70, D32, and D42; a single study identified cerebral meningioma occurrence through histopathological diagnosis [20].

### 3.3. Quality Assessment

Nearly all studies received a Yes across all ten questions (Table 2). The comparability of the cases and controls in the Wahyuhadi study [20] was uncertain as controls were patients who underwent a head CT; as a result, there is a potential for information bias if the dMPA exposure status of the controls is related to the medical condition that was the reason for seeking clinical care. In addition, there was no apparent adjustment for confounders in logistic models. The reliability of dMPA exposure measurement in Xiao [24] was uncertain as it was based on HCPCS and ICD-10 CM codes for an encounter for injectable contraceptive rather than through use of pharmaceutical claims or self-report in prior studies; however, any bias was likely non-differential between case and controls.

### 3.4. Publication Bias Analysis

**Figure 2:**
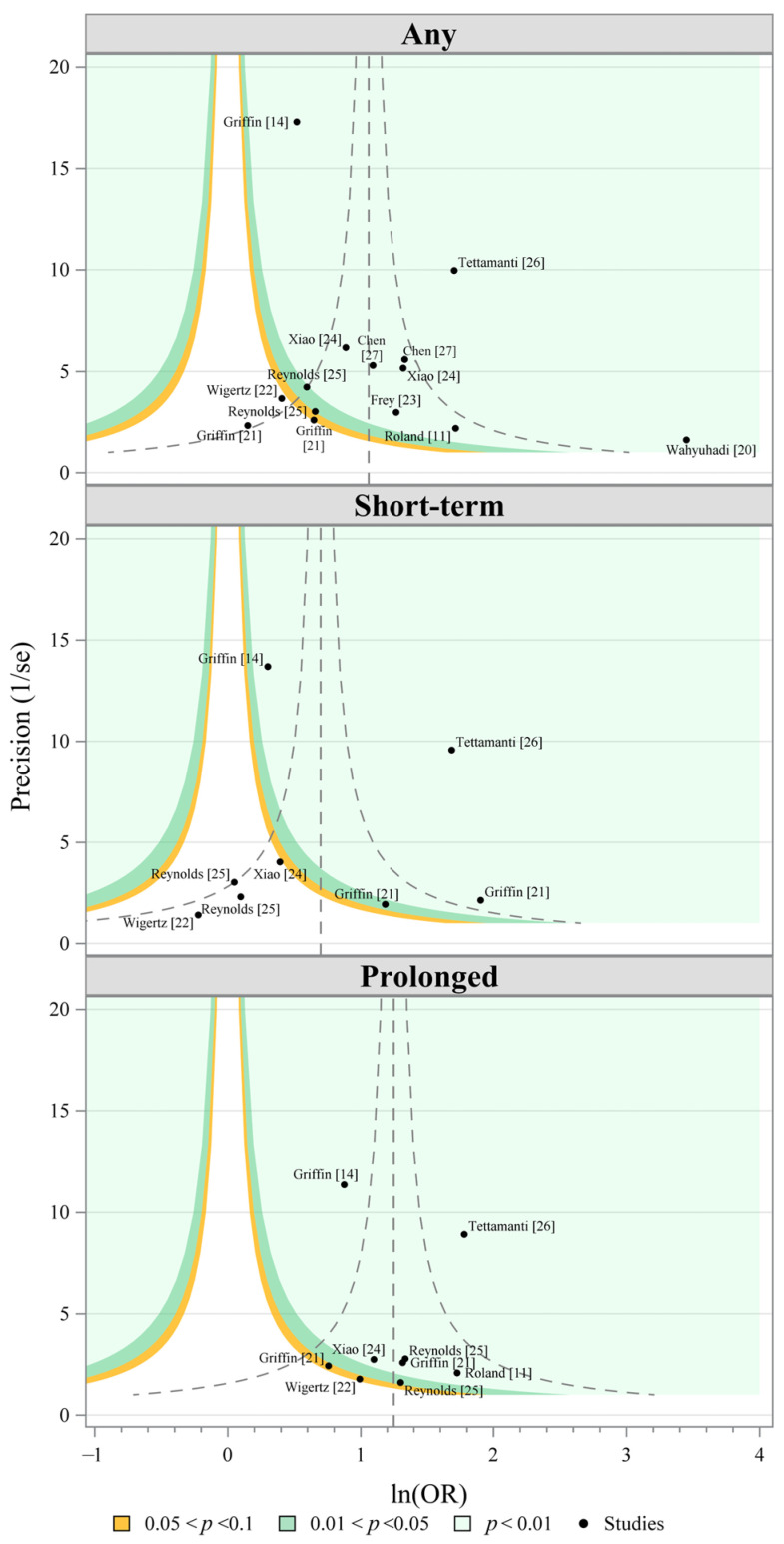
Contoured funnel plots to assess the potential of publication bias by duration of use; short-term: <2 years continuous; prolonged: ≥2 years continuous. The x-axis represents the natural log of the odds ratio and the y-axis the precision of the study, defined as the inverse of the standard error. The contours denote levels of statistical significance ranging from white (*p* > 0.01) to light gray (*p* < 0.01). The vertical dashed line denotes the pooled odds ratio and curved dashed lines the interpolated 95% confidence intervals. Publication bias is suggested by asymmetry of studies on either side of the vertical dashed line. Egger’s regression test of asymmetry suggests no evidence of asymmetry: Any exposure *p* = 0.2590; short-term exposure: *p* = 0.6434; prolonged exposure: *p* = 0.9526. The included studies were Wigertz (2006) [22]. Wahyuhadi (2018) [20], Roland (2024) [11], Griffin (2024) [14], Griffin (2025) [21], Frey (2025) [23], Xiao (2025) [24], Reynolds (2025) [25], Tettamanti (2025) [26], and Chen (2026) [27].

Examining the contoured funnel plots, there is potential asymmetry for studies assessing short-term dMPA use with potential missing studies with stronger associations that are significant, resulting in a pooled short-term association that may be weaker than the true pooled association (Figure 2); however, as the imputed missing studies would be towards the stronger, significant associations, it is likely that the exclusion is not due to publication bias based on statistical significance [28]. Further, results of Egger’s regression test suggest no statistical evidence of asymmetry with observed non-significant *p*-values for any exposure (*p* = 0.2590), short-term exposure (*p* = 0.6434), and prolonged exposure (*p* = 0.9526).

### 3.5. Pooled Analysis and Heterogeneity Analysis

**Figure 3:**
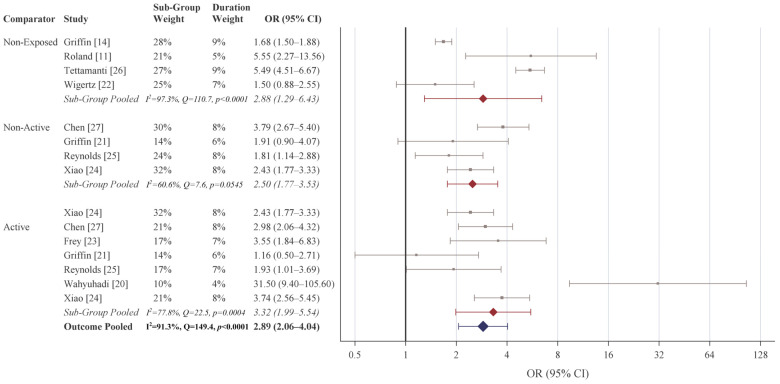
Meta-analysis of the association between medroxyprogesterone acetate and cerebral meningioma for any exposure to depot medroxyprogesterone acetate. Study-specific odds ratios in gray; sub-group pooled odds ratios in red; overall exposure duration odds ratio in blue. Odds ratios based on random-effects estimation. The included studies were Wigertz (2006) [22], Wahyuhadi (2018) [20], Roland (2024) [11], Griffin (2024) [14], Griffin (2025) [21], Frey (2025) [23], Xiao (2025) [24], Reynolds (2025) [25], Tettamanti (2025) [26], and Chen (2026) [27].

**Figure 4:**
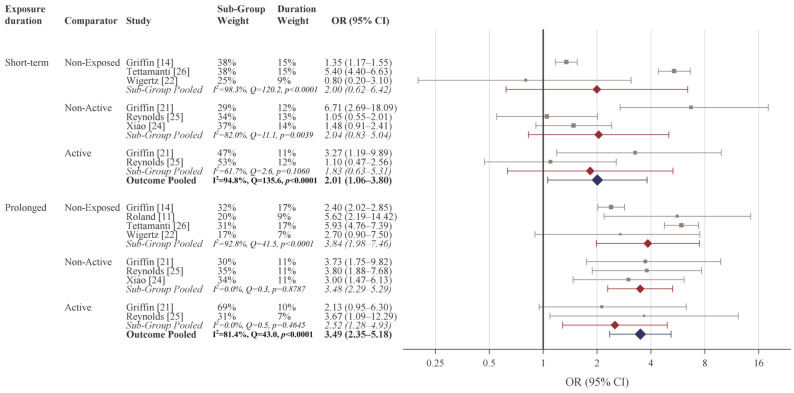
Meta-analysis of the association between medroxyprogesterone acetate and cerebral meningioma by duration of depot medroxyprogesterone acetate exposure and type of comparator studied. Short-term exposure defined as <2 years; prolonged exposure defined as ≥2 years use. Study-specific odds ratios in gray; sub-group pooled odds ratios in red; overall exposure duration odds ratio in blue. Short-term use: <2 years continuous use; prolonged: ≥2 years continuous use. The included studies were Wigertz (2006) [22], Roland (2024) [11], Griffin (2024) [14], Griffin (2025) [21], Xiao (2025) [24], Reynolds (2025) [25], Tettamanti (2025) [26].

Including all associations across the studies, the pooled OR was 2.78 (2.20–3.52) (Table 3). For any exposure to dMPA, the pooled association was OR 2.89 (95% CI 2.06–4.04), with the strongest association observed for comparisons to an active comparator (OR 4.43, 95% CI 1.99–5.54) (Figure 3). By specific exposure duration, the strongest association was observed for prolonged dMPA exposure (pooled OR 3.49, 95% CI 2.35–5.18), particularly for comparisons to a non-exposed comparator (OR 3.84, 95% CI 1.98–7.46) with a weaker, yet still statistically significant, association compared to an active comparator (OR 2.52, 95% CI 1.28–4.93) (Figure 4). The overall pooled association for short-term exposure was weakest among the three duration categories yet still statistically significant (OR 2.01, 95% CI 1.06–3.80) with no significant effects observed among the three comparison sub-group specific pooled associations.

In sensitivity analyses by duration category and including (a) the weakest association from each study and (b) the strongest association from each study, pooled associations were similar to the above-reported associations for any exposure (weakest OR pooled: 2.95, 95% CI 1.92–4.51; strongest OR pooled: 3.37, 95% CI 2.15–5.29), short-term exposure (weakest OR pooled: 1.84, 95% CI 0.0.89–3.81; strongest OR pooled: 2.09, 95% CI 0.99–4.41), and prolonged exposure (weakest OR pooled: 3.41, 95% CI 2.13–5.46; strongest OR pooled: 3.69, 95% CI 2.38–5.74).

**Figure A1:**
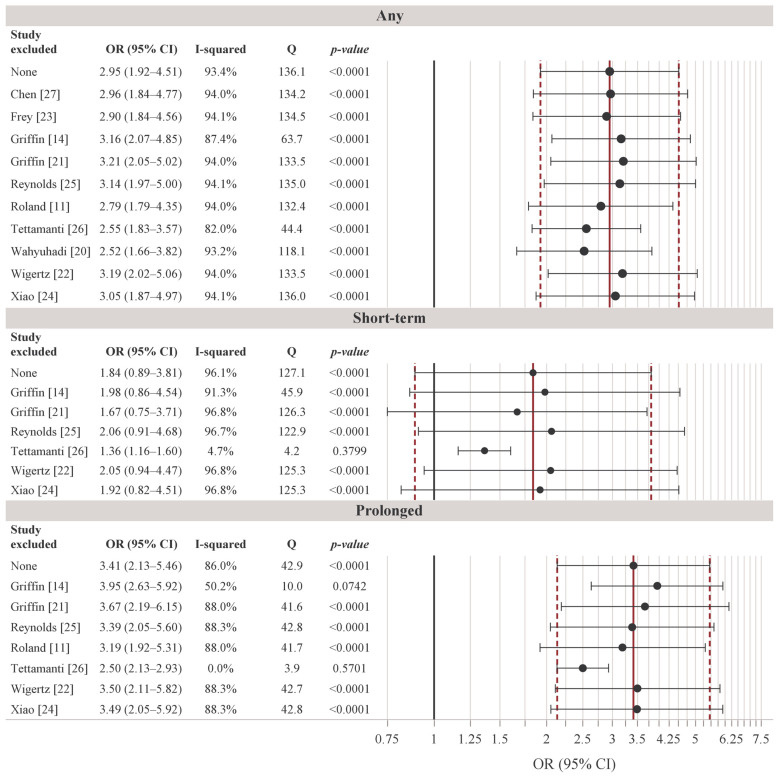
Forest plot of leave-one-out analysis utilizing weakest associations from each study by for each exposure duration. Black vertical line is the null value of an odds ratio; red solid line is the pooled odds ratio; and red dashed lines are the 95% confidence intervals of the pooled odds ratio. The included studies were Wigertz (2006) [22]. Wahyuhadi (2018) [20], Roland (2024) [11], Griffin (2024) [14], Griffin (2025) [21], Frey (2025) [23], Xiao (2025) [24], Reynolds (2025) [25], Tettamanti (2025) [26], and Chen (2026) [27].

There was significant heterogeneity for the global effect (I^2^ = 92.2%, Q = 319.5, *p* < 0.0001); among studies that used any exposure to dMPA (I^2^ = 91.3%, Q = 149.407, *p* < 0.0001); among studies assessing short-term exposure (I^2^ = 94.8%, Q = 135.6, *p* < 0.0001); and among studies assessing prolonged exposure associations (I^2^ = 81.4%, Q = 43.0, *p* < 0.0001). In leave-one-out analyses (Figure A1), the association for any exposure remained relatively stable with a low association of OR 2.52 (95% CI 1.66–3.82) estimated odds ratios based on random-effects estimation after excluding Wahyuhadi [20] and a high association when excluding Wigertz [22] (OR 3.19, 95% CI 2.02–5.06). For both short-term exposure and prolonged exposure, exclusion of Tettamanti [24] resulted in no heterogeneity (short-term: I^2^ = 4.7%, Q = 4.2, p = 0.3799; prolonged: I^2^ = 0.0%, Q = 3.9, p = 0.5701) and the weakest associations (short-term: OR 1.36, 95% CI 1.16–1.60; prolonged: OR 2.50 (95% CI 2.13–2.93)). The strongest association for short-term exposure was observed when excluding Reynolds [25] (OR 2.06, 95% CI 0.91–4.68) and for prolonged exposure when excluding Griffin (2024) [14] (OR 3.95, 95% CI 2.63–5.92).

**Figure A2:**
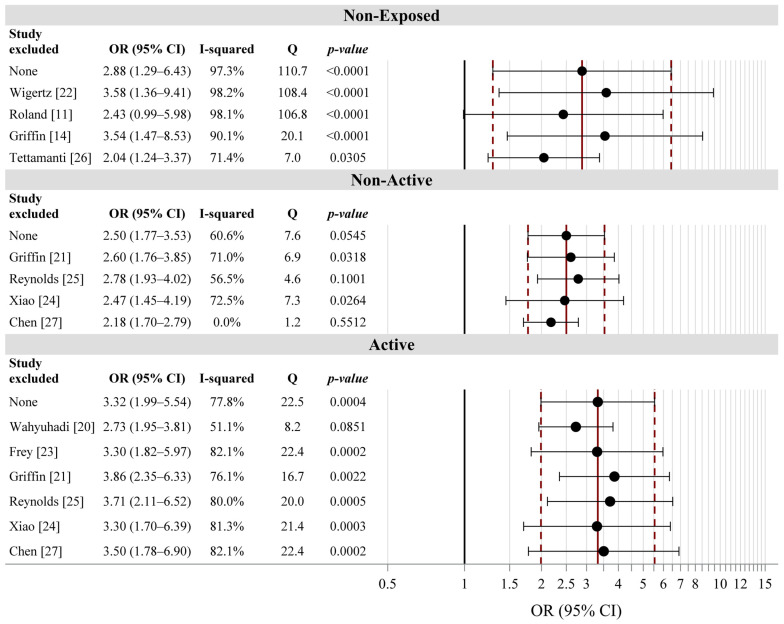
Forest plot of leave-one-out analysis results for any exposure to depot medroxyprogesterone acetate by type of comparator. Black vertical line is the null value of an odds ratio; red solid line is the pooled odds ratio; and red dashed lines are the 95% confidence intervals of the pooled odds ratio. *p*-value is for the Q-statistic with a *p*-value < 0.05 denoting significant heterogeneity. The included studies were Wigertz (2006) [22], Wahyuhadi (2018) [20], Roland (2024) [11], Griffin (2024) [14], Griffin (2025) [21], Frey (2025) [23], Xiao (2025) [24], Reynolds (2025) [25], Tettamanti (2025) [26], and Chen (2026) [27].

**Figure A3:**
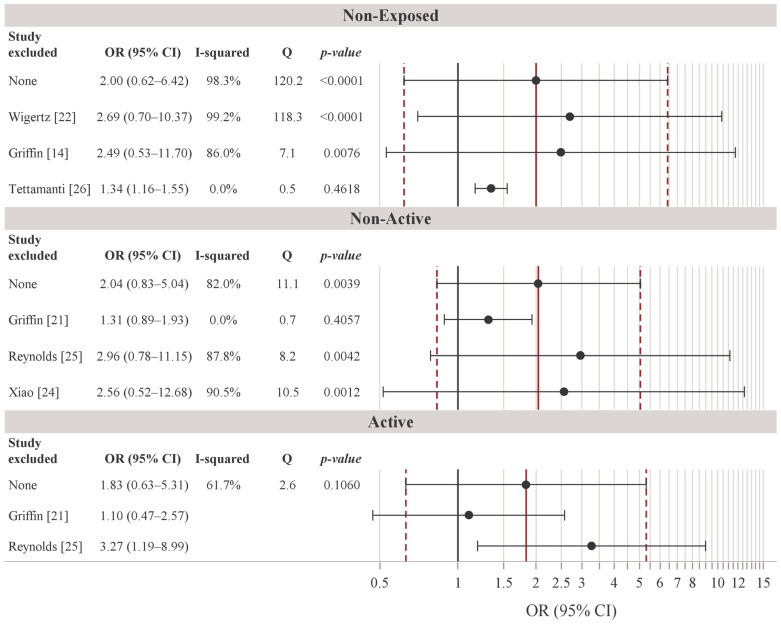
Forest plot of leave-one-out analysis results for short-term exposure to depot medroxyprogesterone acetate by type of comparator. Black vertical line is the null value of an odds ratio; red solid line is the pooled odds ratio; and red dashed lines are the 95% confidence intervals of the pooled odds ratio. *p*-value is for the Q-statistic with a *p*-value < 0.05 denoting significant heterogeneity. The included studies were Wigertz (2006) [22], Griffin (2024) [14], Griffin (2025) [21], Xiao (2025) [24], Reynolds (2025) [25], and Tettamanti (2025) [26].

**Figure A4:**
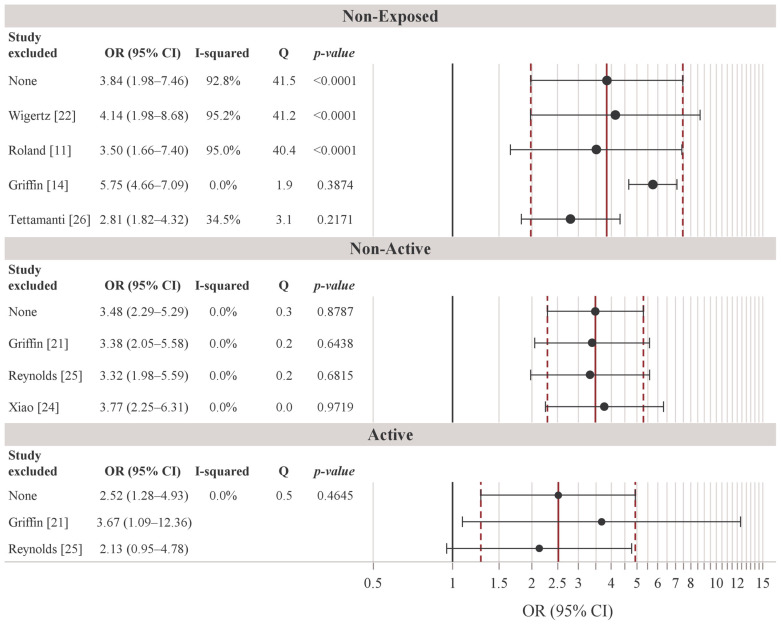
Forest plot of leave-one-out analysis results for prolonged exposure to depot medroxyprogesterone acetate by type of comparator. Black vertical line is the null value of an odds ratio; red solid line is the pooled odds ratio; and red dashed lines are the 95% confidence intervals of the pooled odds ratio. *p*-value is for the Q-statistic with a *p*-value < 0.05 denoting significant heterogeneity. The included studies were Wigertz (2006) [22], Roland (2024) [11], Griffin (2024) [14], Griffin (2025) [21], Xiao (2025) [24], Reynolds (2025) [25], and Tettamanti (2025) [26].

When performing the leave-one-out analysis by combinations of exposure duration and comparator type, associations remained similar in strength for the any exposure categorization (Figure A2). Among short-term exposure associations (Figure A3), the pooled comparison to a non-active comparator was lowest when excluding Griffin (2025) [21] (OR 1.31, 95% CI 0.89–1.93, I^2^ = 0.0%, Q = 0.7, *p* = 0.4057), and pooled comparisons to non-exposed subjects were lowest when excluding Tettamani [26] (OR 1.34, 95% CI 1.16–1.55, I^2^ = 0.0%, Q = 0.5, *p* = 0.4645). Among prolonged exposure comparisons (Figure A4), there was no heterogeneity among comparisons to active or non-active comparator subjects; the significant heterogeneity for comparisons to non-exposed subjects was removed when excluding either Griffin 2024 [14], which resulted in the strongest pooled association (OR 5.75, 9% CI 4.66–7.09, I^2^ = 0.0%, Q = 1.9 *p* = 0.3874) or Tettamani [26], which resulted in the weakest pooled association (OR 2.81, 95% CI 1.83–4.32, I^2^ = 34.5%, Q = 3.1, *p* = 0.2171).

### 3.6. Quality of Evidence

GRADE assessments for reviews based on observational research begin with a low grade of evidence. Based on the funnel plots and Egger’s regression test, there is no concern for publication bias (Table A1). Further, as all studies were observational and mostly based on registry or claims data, there is no concern noted for risk of bias due to selection or information bias. No concern was noted for imprecision as a majority of associations were statistically significant and were based on studies using large population registry or medical claims data. The heterogeneity was driven by select studies, the exclusion of which had limited effect on the inference of the pooled effect; as a result, no concern of inconsistency was noted.

Regarding residual confounding, as observational studies do not have a controlled, randomized aspect to the design, there is potential for residual confounding in the included studies; however, per Balshem [16] and Prasad [17], concerns for risk of bias are not noted if the confounding effect is towards the null. For the included studies, there is no reason to believe that residual confounding in the included studies, a vast majority of which are based on registry or claims data, would be differential by cerebral meningioma status or depot medroxyprogesterone acetate exposure status. As a result, the non-differential confounding bias results in reported associations being biased towards the null, and accounting for the confounding would result in stronger associations; thus, no concern for risk of bias is present.

That said, due to the difference in the source populations and the significant, high heterogeneity, a very serious concern of indirectness was noted, downgrading the GRADE by two (Table 3). From the pooled analysis, the GRADE evidence quality was upgraded due to a large, pooled effect size (i.e., >OR 2.00) and presence of a dose–response gradient (prolonged association stronger than short-term association). This yielded a final quality of evidence rating of moderate, suggesting that the true estimate is near the reported pool observations, though a true effect different than the reported pooled association cannot be ruled out. Based on the overall pooled OR of 2.78 (2.20–3.52) and an incidence of 12.5 per 100,000 persons from the prior literature [19], the estimated risk of cerebral meningioma among those with dMPA exposure is 34.8 (95% CI 27.5–44.0) per 100,000, a risk difference of 22.3 (95% CI 15.0–31.5) per 100,000.

## 4. Discussion

The results of this meta-analysis confirm associations from the previous literature. Specifically, the current study reported an over two-fold association between exposure to dMPA and cerebral meningioma. This association is strongest for prolonged use (i.e., at least two years of continuous use) rather than short-term use (i.e., fewer than two years), though it is present at a significantly increased effect for any exposure and short-term exposure. Further, the observed pooled association is persistent whether the comparison group comprises those with either no exposure to hormonal contraceptives or users of non-dMPA hormonal contraception. This study adds to the published literature an assessment of the quality of evidence in addition to potential biases such as publication bias of studies published from inception through February 2026; in addition, the study is the first to provide a meta-analysis by dMPA exposure duration and comparator type, providing a more thorough examination of pooled associations of dMPA and cerebral meningioma. It is worth noting that over two-thirds of the 32 associations included in the current analysis were statistically significant. Of the nine associations that were not significant, three were from the same study that was the earliest performed and had the second smallest sample size [22] behind Wahyuhadi [20]. Further, of the studies examining prolonged dMPA use, only two reported non-significant associations. Both were based on smaller case-control studies [21,22] but reported strengths of association similar to the other studies.

Though the physiological mechanism underlying the association between dMPA and cerebral meningiomas is not fully described, there are potential mechanisms that have been discussed. Progestin-associated cerebral meningiomas have been reported to occur more often among the skull base [29], a location that has been associated with higher rates of mutations to the *PIK3CA* gene [30]. The *PIK3CA* gene is believed to be involved in the apoptosis of meningeal cells, and the mutations result in an activation of the *PI3K/AKT/mTOR* pathway. This then results in a cascade of events that lead to decreased apoptosis [31], formation of tumors [32], and tumor progression [33].This proposed mechanism could potentially explain the observed pattern in the current meta-analysis that prolonged dMPA exposures are most strongly associated with cerebral meningioma as the longer duration of exposure potentially results in an increased chance of *PIK3CA* mutation due to longer exposure time.

These results should be viewed in light of the limitations of the current search methodology, mainly that only three services (PubMed, Embase, Web of Science) were reviewed; thus, it is possible that studies were excluded from the review if published in a journal not indexed in one of the three services. However, research has suggested that as few as two services is sufficient to include related literature on a given topic [34] while another suggests the three services used in the current analysis allow for appropriate coverage of literature on a topic [35]. In addition, the review was limited to observational research studies; however, in the search, no studies based on clinical trials were identified. As a final limitation, there was high heterogeneity in the current meta-analysis that was particularly driven—as evidenced in the leave-one-out-analysis—by Tettamanti [26] and Griffin (2025) [21]. Despite the high heterogeneity, there was minimal change in the strength and inferences of the associations during the leave-one-out analysis, lending credence to the robustness of the pooled associations despite the high heterogeneity.

It is also important to place these studies in the context of their strengths and limitations. First, nearly all studies utilized pharmaceutical claims or medical record reviews to determine dMPA exposure. While this type of medication exposure ascertainment can lead to misclassification bias if the subject does not truly take the medication, this bias is likely not present for dMPA, which is a one-time injection given every three months. Thus, the claims and medical record data accurately present whether the person was exposed. That said, it is possible that the injection is not reported in the claims or medical record data, resulting in the person being incorrectly misclassified as unexposed; however, this would result in a bias towards the null as it is unlikely to be differential between cerebral meningioma case and controls and, if it were to occur, would result in an overestimate of cerebral meningioma in the unexposed group, decreasing the strength of association. As another limitation, since the data for most of the studies were derived from a hospital or medical claims data, controls may not be representative of the exposure prevalence of the general population. The effects of this limitation, however, are likely minimal as the claims data encompasses inpatient and outpatient claims across multiple medical facilities. Finally, the prevalence of dMPA exposure was low among cases in multiple studies, which could affect statistical power and could be due to the exposure prevalence of the studies not being representative of the general population; however, there is no reason to suspect the non-representativeness to be differential by case or non-case status.

The results of this meta-analysis have important clinical implications; however, prior to discussion of the clinical impact, one must take into consideration the potential causation of associations derived from observational studies. Specifically, one must consider strength of association, consistency of association, dose–response relationship, temporality of exposure, and specificity of association. The first three are described within the GRADE methodology. In the current analysis, dMPA exposure was deemed a moderate quality due in part to the strong association, the consistency of the association, and the dose–response relationship. The latter two criteria are described by Bradford Hill [36] and often used to aid in epidemiological research in the determination of potential causality of an association. The studies included in this analysis all meet the temporality criteria as the use of data from prior cohorts and administrative data allowed researchers to determine the timing of dMPA exposure relative to the diagnosis of cerebral meningioma. Regarding specificity, null effects have been reported for dMPA exposure association and spinal meningioma and oral MPA exposure for cerebral meningioma or spinal meningioma [20], suggesting that the exposure increases the risk only for cerebral meningioma and for dMPA. Taken together, the associations reported in the current analysis provide evidence to support a potential causal association between dMPA and cerebral meningioma; however, causation is not a certainty given the limitations of observational research regarding confounding and selection bias, and, further, more rigorous research is needed.

## 5. Conclusions

Despite the stated limitations, the selected studies provide evidence that dMPA is associated with the diagnosis of cerebral meningioma. The source of the study populations of the studies has been varied—including random selection of the source general population, cancer patients treated at a medical center, and enrollees in a public or private insurance—yet the reported associations remain consistent across the studies. Further, the studies have observed that the association is specific to dMPA (reported associations for oral MPA have been null), specific to cerebral meningioma (reported associations for spinal meningiomas have been null), and increase in strength with longer duration of use, particularly an over three-fold increased association for prolonged dMPA use. The results of the current analysis and review provide evidence that the association between dMPA and cerebral meningioma could be causative; however, further research is warranted on the topic of injectable MPA and the association with meningioma. In particular, it would be of interest to examine the association between dMPA and meningioma WHO grade as the literature on that topic is lacking as of the current date. In addition, research is needed on the progression of dMPA-related meningiomas (in order to determine, for example, the proportion of time surgical treatment is needed) as well as long-term cognitive and quality-of-life outcomes of females with MPA-related meningiomas.

Further, while keeping in mind the discussion of the concept of causation in epidemiology, the results of this meta-analysis suggest that, first and foremost, clinicians should discuss with their patients the potential risk of meningioma associated with dMPA. The American College of Obstetricians and Gynecologists recommends a shared decision-making approach in which clinicians provide clear explanations of the evidence of the risks associated with contraception use, allowing the patient to make an informed decision on whether to use the contraceptive [37]. Part of that discussion should include the recent FDA requirement that dMPA should include a warning regarding the risk of meningioma [38]. A recent review article of dMPA and cerebral meningioma provided further suggestions, including advising against the use of dMPA for more than two years and for women who use dMPA to monitor for signs of cerebral meningioma including headaches, vision changes, or seizures [12]. Research has reported that progestin-related meningiomas decrease in size following discontinuation of non-MPA progestins including cyproterone acetate, chlormadinone acetate, and nomegestrol acetate [39,40,41,42,43]. Finally, there are other contraceptives within the progestogen class such as levonorgestrel or desogestrel that have been reported to not be associated with a risk of cerebral meningioma [44,45]. Clinicians should discuss the use of other progestin contraceptives if they are available to the patient; however, caution should be used in the selection of an alternative progestin as research has reported an increased risk of meningioma with the use of cyproterone acetate [40], chlormadinone acetate [46], and nomegestrol acetate [47].

## Figures and Tables

**Table 1:** Characteristics of studies selected for final analysis.

	Location, Source, Study Design, and Years Included	Participants (n)	Matching Criteria	Exposed (n)	dMPA Definition	Comparator Type	Confounders
Wigertz (2006) [22]	Sweden; INTERPHONE study; unmatched case-control; 2000–2002	178 cases; 323 controls; ages 20–69 y	Age	30 cases, 43 controls	Self-reported; ever-use and duration of use	Non-users of hormone contraceptives	Age, residential area, education, and parity
Wahyuhadi (2018) [20]	Patients treated at Dr. Soetomo Hospital in Surabaya, Indonesia; unmatched case-control; 2012–2013	101 cases; 101 controls; ages 20–65 y	N/A	84 cases, 14 controls	Medical record review; ever-use	Non-users of hormone contraceptives	None
Roland (2024) [11]	French national health data system (Système National des Données de Santé [SNDS]); national case-control; 2009–2018	18,601 cases; 90,305 controls; all ages	Year of birth; area of residence	9 cases, 11 controls	National health record data; WHO’s anatomical, therapeutic, and chemical (ATC) classification (G03AC06, L02AB02); ever-use and duration of use	Non-users of hormone contraceptives	None
Griffin (2024) [14]	United States; IBM MarketScan database; matched case-control; 2006–2022	117,503 cases; 1,072,907 controls; ages ≥ 18 y	Age ± 1 year and exact year of enrollment	480 cases, 2626 controls	Pharmacy claims data; ever-use; duration of use	Non-users of hormone contraceptives	Age and unweighted Elixhauser comorbidity score
Griffin (2025) [21]	United States; Patients treated at academic medical center; matched case-control; 2015–2024	241 cases; cancer controls matched 3:1 via bootstrapping; ages ≥ 18 y	Age ± 5 years and a cancer diagnosis within three months of the case diagnosis date	7 cases; mean of 1% of controls across bootstrapped cycles	Medical record review; ever-use; duration of use	Active comparator; non-users of hormone contraceptives	Age, race, and urban/rural classification of residential ZIP code, insurance type, and unweighted Elixhauser comorbidity score
Frey (2025) [23]	United States; PharMetrics^®^ Plus for Academics Database; nested case-control; 2006–2020	212 cases; 848 controls; age range not stated	Age ± 1 year and calendar time	21 cases, 33 controls	Pharmacy claims data; duration of use	Active comparator	Age, obesity, previous contraceptive use, previous radiation therapy
Xiao (2025) [24]	United States; TriNetX; cohort; 2004–2024	88,667 exposed; 88,667 propensity-matched unexposed; age range not stated	Age at inclusion, race, ethnicity, history of pregnancy, history of breast cancer, neurofibromatosis, history of radiation exposure, and body mass index	88,667 total exposed, of which 131 were diagnosed with meningioma	HCPCS J1050 (dMPA injection); ICD-10 codes Z30.42 or Z30.013 for encounter for injectable contraceptive ever-use and duration of use	Active comparator; non-users of hormone contraceptives	None
Reynolds (2025) [25]	United States; Medicaid; matched case-control; 2010–2023	469 cases; 4690 controls; ages 18–55 y	Age ± 1 year and year of Medicaid enrollment; and calendar time	29 cases, 182 controls	Pharmacy claims data; ever-use; duration of use	Active comparator; non-users of hormone contraceptives	Age, race, and number of Elixhauser comorbidities
Tettamanti (2025) [26]	Swedish cancer registry/ population register; matched case-control; 2007–2015	1055 cases; 21,000 controls; ages ≥ 20 y	Birth year and county of residence at case index date	186 cases, 853 controls	National health record data; ATC code G03AC06; ever-use; number of prescriptions	Non-users of hormone contraceptives	Marital status, educational level, income, parity, history of diseases of the circulatory system, and family history of breast cancer and central nervous system tumors
Chen (2026) [27]	United States; Merative Marketscan; matched case-control; 2005–2019	1218 cases; 12,172 controls; ages 15–42 at cohort entry	Age, cohort entry date, and follow-up time	46 cases, 132 controls	Pharmacy claims data; ever-use	Non-users of hormone contraceptives; active comparator	Age at cohort entry, obesity, hypertension, obstructive sleep apnea, diabetes mellitus type 2, breast cancer, and uterine fibroids

**Table 2:** JBI critical appraisal of selected studies.

	Groups Comparably Selected	Appropriate Matching	Same Criteria for Cases/Controls	Reliable Exposure Measurement	Consistent Exposure Measurement	Confounding Factors Identified	Stated Adjustment Methods	Reliable/Valid Outcome Measurement	Exposure Period Long Enough	Statistical Analysis Appropriate
Wigertz (2006) [22]	Yes	Not applicable	Yes	Yes	Yes	Yes	Yes	Yes	Yes	Yes
Wahyuhadi (2018) [20]	Uncertain	Not applicable	No	Yes	Yes	Yes	No	Yes	Yes	Yes
Roland (2024) [11]	Yes	Yes	Yes	Yes	Yes	Yes	Yes	Yes	Yes	Yes
Griffin (2024) [14]	Yes	Yes	Yes	Yes	Yes	Yes	Yes	Yes	Yes	Yes
Griffin (2025) [21]	Yes	Yes	Yes	Yes	Yes	Yes	Yes	Yes	Yes	Yes
Frey (2025) [23]	Yes	Yes	Yes	Yes	Yes	Yes	Yes	Yes	Yes	Yes
Xiao (2025) [24]	Yes	Yes	Yes	Uncertain	Yes	Yes	Yes	Yes	Yes	Yes
Reynolds (2025) [25]	Yes	Yes	Yes	Yes	Yes	Yes	Yes	Yes	Yes	Yes
Tettamanti(2025) [26]	Yes	Yes	Yes	Yes	Yes	Yes	Yes	Yes	Yes	Yes
Chen (2026) [27]	Yes	Yes	Yes	Yes	Yes	Yes	Yes	Yes	Yes	Yes

**Table 3:** Summary of findings table for Grading of Recommendations Assessment, Development, and Evaluation (GRADE) ratings for the association between depot medroxyprogesterone acetate (dMPA) exposure and cerebral meningioma.

	Absolute Effect (per 100,000)				
	Without * dMPA	With dMPA (95% CI)	Relative Effect Odds Ratio (95% CI)	Number of Studies	Certainty of the Evidence †
Outcome					
Cerebral meningioma	12.5	34.8 (27.5–44.0)	2.78 2.20–3.52	10	⊕⊕⊕◯ Moderate

* The risk of cerebral meningioma without dMPA exposure based on the age-adjusted risk reported for females aged 35–54 years by Cao et al. (2023) [19]. † Downgraded due to inconsistency of study population and exposure prevalence of dMPA. ⊕ Denotes a positive scale-point on a four-point scale of the quality of evidence. ◯ Denotes a negative scale-point on a four-point scale of the quality of evidence.

## Data Availability

Data collected from included studies as well as analytic code and materials are not publicly available.

## Supplementary Materials

The following supporting information can be downloaded at: https://www.mdpi.com/article/10.3390/cancers18081252/s1, PRISMA 2020 Checklist. Reference [48] is cited in the Supplementary Materials.

## Abbreviations

The following abbreviations are used in this manuscript:

GRADE	Grading of Recommendations Assessment, Development, and Evaluation
dMPA	Depot Medroxyprogesterone Acetate
OR	Odds Ratio
95% CI	95% Confidence Interval

## Appendix A

**Table A1:** Grading of Recommendations Assessment, Development, and Evaluation (GRADE) ratings for the association between depot medroxyprogesterone acetate and cerebral meningioma.

GRADE Criteria	Rating	Notes
Observational study evidence quality	⊕⊕◯◯ Low	Starting GRADE evidence rating for observational research
Quality of evidence assessment		
Risk of bias	No	Included studies utilized data from administrative datasets or previously established cohorts
Inconsistency	No	Heterogeneity is high, but associations similar in leave-one-out analysis
Indirectness	Very serious (−2)	Study populations based on members of an insurance group, whether private or public, or from different countries. Cerebral meningioma outcome definitions slightly different across studies.
Imprecision	No	67% of associations were statistically significant with lower SEs
Publication bias	Unlikely	No apparent patterns of asymmetry in funnel plots; tests of asymmetry not significant
Large effect	Large (+1)	Overall pooled effect OR > 2.00
Dose–response gradient	Yes (+1)	Over 3-fold for prolonged use, weaker association for short-term use
Negative residual confounding	Yes (+1)	Confounding likely non-differential, resulting in bias towards the null and currently reported associations being weaker than the true association
Overall quality of evidence rating	⊕⊕⊕◯ Moderate	

⊕ Denotes a positive scale-point on a four-point scale of the quality of evidence. ◯ Denotes a negative scale-point on a four-point scale of the quality of evidence.
